# Are the epidemic prevention facilities effective? How cities should choose epidemic prevention facilities: Taking Wuhan as an example

**DOI:** 10.3389/fpubh.2023.1125301

**Published:** 2023-03-30

**Authors:** Lei Hua, Rong Ran, Zhengxing Ni

**Affiliations:** School of Public Policy and Administration, Chongqing University, Chongqing, China

**Keywords:** COVID-19, urban safety, epidemic prevention facilities, accessibility analyticity, breakpoint regression

## Abstract

The COVID-19 pandemic highlighted the limitations of urban public health emergency response capabilities. Taking Wuhan as an example, this study used breakpoint regression, kernel density analysis, overlay analysis, and accessibility analysis from Stata and ArcGIS, and divided epidemic prevention facilities into the basic epidemic prevention facilities (hospitals), and the emergency epidemic prevention facilities (mobile cabin hospitals) for further analysis. The results showed that over 70% of the basic epidemic prevention facilities in Wuhan were located in high density population areas. On the contrary, most of the emergency epidemic prevention facilities were located in low density population areas. The local treatment effect of the implementation of the emergency epidemic prevention facility policy is about 1, indicating that there was a significant impact of emergency epidemic prevention facilities on outbreak control, which passed the bandwidth test. What’s more, the analysis of the accessibility of residential points revealed that more than 67.3% of people from the residential points could arrive at the epidemic prevention facilities within 15 min, and only 0.1% of them took more than 20 min to arrive. Therefore, the epidemic prevention facilities can effectively curb the spread of the epidemic, and people from residential areas can quickly get there. This study summarized the spatial characteristics of epidemic prevention facilities in Wuhan and analyzed the importance of them, thus providing a new perspective for future research on upgrading the city’s comprehensive disaster prevention system.

## 1. Introduction

In recent decades, more and more cities around the world have been affected by urban disasters, so urban public safety issues have become a big obstacle for urban development (1, 2). The suddenness of natural disasters, the frequency of man-made disasters, and the proliferation of public health and safety events have led to a significant increase in the types and contents of disasters, which needs to be considered for urban disaster prevention (3–5). The current high population density, high population mobility, delayed infrastructure construction, chaotic structural layout, and large building vulnerability in cities have brought a severe test to the safety of cities (6, 7). How to solve the weak foundation of urban disaster prevention, how to improve emergency response capability, and how to generally improve the comprehensive disaster prevention capability of cities have become a tough task in the current era (8–10). It has become a major issue worldwide since the first reported detection of the COVID-19 outbreak in December 2019 (11, 12). As of September 15^th^, 2021, the COVID-19 epidemic has spread to 212 countries with a cumulative amount of 226,777,522 infections and 4,663,829 deaths, making it a public health event that threatens the whole world. As a public health emergency, COVID-19 is characterized by its suddenness, complexity, devastation, and unpredictability (13, 14). Many countries have adopted various prevention and control policies to control the increase of infected people, such as limiting traveling, maintaining social distance, and restricting large gatherings (15). In addition, many countries have introduced regulations that require isolation of infected individuals and quarantine of contacts (16, 17). Existing studies suggest that isolating the source of infection is a beneficial measure to control the spread of the epidemic, but how to isolate it? In which way? Where to isolate? So it has become a question that needs to be answered urgently (18). Therefore, timely identification, effective control, and scientific measures to deal with public health emergencies are related to the safety of people’s lives and social stability, which is not only a great challenge to urban public safety, but also a vital test of the city’s comprehensive disaster prevention and mitigation capabilities.

At present, although an increasing number of international studies focus on the impact of natural and accidental disasters on urban public safety and urban disaster prevention and mitigation, there is a lack of research on urban public health emergencies (19). In fact, epidemic prevention facilities are rarely mentioned in comprehensive disaster prevention and mitigation plans for cities around the world (20, 21). Most of the studies about urban disaster facilities focus on assessing the ability of cities to cope with natural disasters from the perspectives of floods, fires, and earthquakes, combing with population data, urban infrastructure, social factors and so on (7, 22–25). Existing studies indicate the characteristics and development trends of global urban disasters and major emergencies, comparing the advantages and disadvantages of urban disaster prevention facilities and conducting a macroscopic assessment of urban emergency measures to optimize the disaster prevention planning (26–28). Tracey et al. conducted a comprehensive evaluation of different aspects of infrastructure layout such as disaster response capacity impact and vulnerability by constructing an evaluation model and evaluation framework and proposed specific optimization recommendations for urban disaster response construction (29, 30). In addition, there are some studies that focus on the accessibility and location of disaster prevention facilities based on road network data, POI, and demand point data, using classical models such as P-median (31), P-center (32), set coverage (33), and two-step floating catchment area model (34–36). Meanwhile, some scholars have studied the issues of facility location and resource allocation, distribution path, and personnel evacuation in terms of decision-making (37). For example, Ferrer et al. considered a multi-objective optimization problem for location-allocation of post-disaster emergency facilities by designing non-dominated sequential genetic algorithms to obtain optimal solutions under multiple objectives of cost, time, reliability, and fairness (38). Marilene et al. further addressed the emergency facility location and distribution problem by constructing a location-route-coverage model (39); Yahyaei and Bozorgi integrated and optimized the model with shelter location, material distribution, and personnel evacuation as themes (40).

In urban epidemic studies, because most of the studies are based on the spread of epidemics, so there is a lack of research on epidemic prevention facilities and epidemic prevention and control tools (41–43). For example, Vick et al. studied disaster response based on disaster plan development, on-site emergency response capabilities, available supplies and resources, disaster education and training, disaster preparedness funding levels, and disaster preparedness awareness (44). Maharaj et al. explored medical facilities under the background of disasters, established the importance of hospitals in disaster management, and analyzed the conversion of ferries into hospitals to centralize patients in Italian after the COVID-19 epidemic, which had achieved better medical treatment and isolation and had a positive effect on the containment of the epidemic (45–47). He et al. further constructed an epidemic prediction and emergency logistics network optimization model under the background of population movement (48). Means et al. concluded that the rapid isolation of diagnosed patients and cutting off the source of infection are effective ways to reduce the spread of the epidemic in case of an outbreak (49, 50). Compared with the existing studies on comprehensive urban disaster prevention and mitigation and strengthening urban public safety capacity, there is a lack of research on epidemic control and disaster prevention facilities.

Wuhan was the first city in China and the world to take control of the COVID-19 epidemic. The city used rare measures such as lockdown and controls to deal with the spread of the epidemic and was the first city that managed to contain the spread of the COVID-19 epidemic (18, 51). Under the background of COVID-19, this study aims to investigate the spatial distribution of epidemic prevention facilities and its influence on the epidemic in Wuhan, a city that has successfully contained the spread of epidemic. Based on Stata and ArcGIS software, this study uses breakpoint regression, overlay analysis, comparative analysis, and accessibility analysis combined with population spatial distribution data, POI data, and road network data to analyze the spatial layout and accessibility of epidemic prevention facilities in Wuhan, as well as their impact on the epidemic, and assess the rationality of the spatial layout of epidemic prevention facilities and their prevention and control capability. It provides reference and guidance for the future prevention and control of major public health events such as an urban epidemic and presents a more complete urban security system for sustainable urban development.

## 2. Methods

### 2.1. Research area

**Figure 1:**
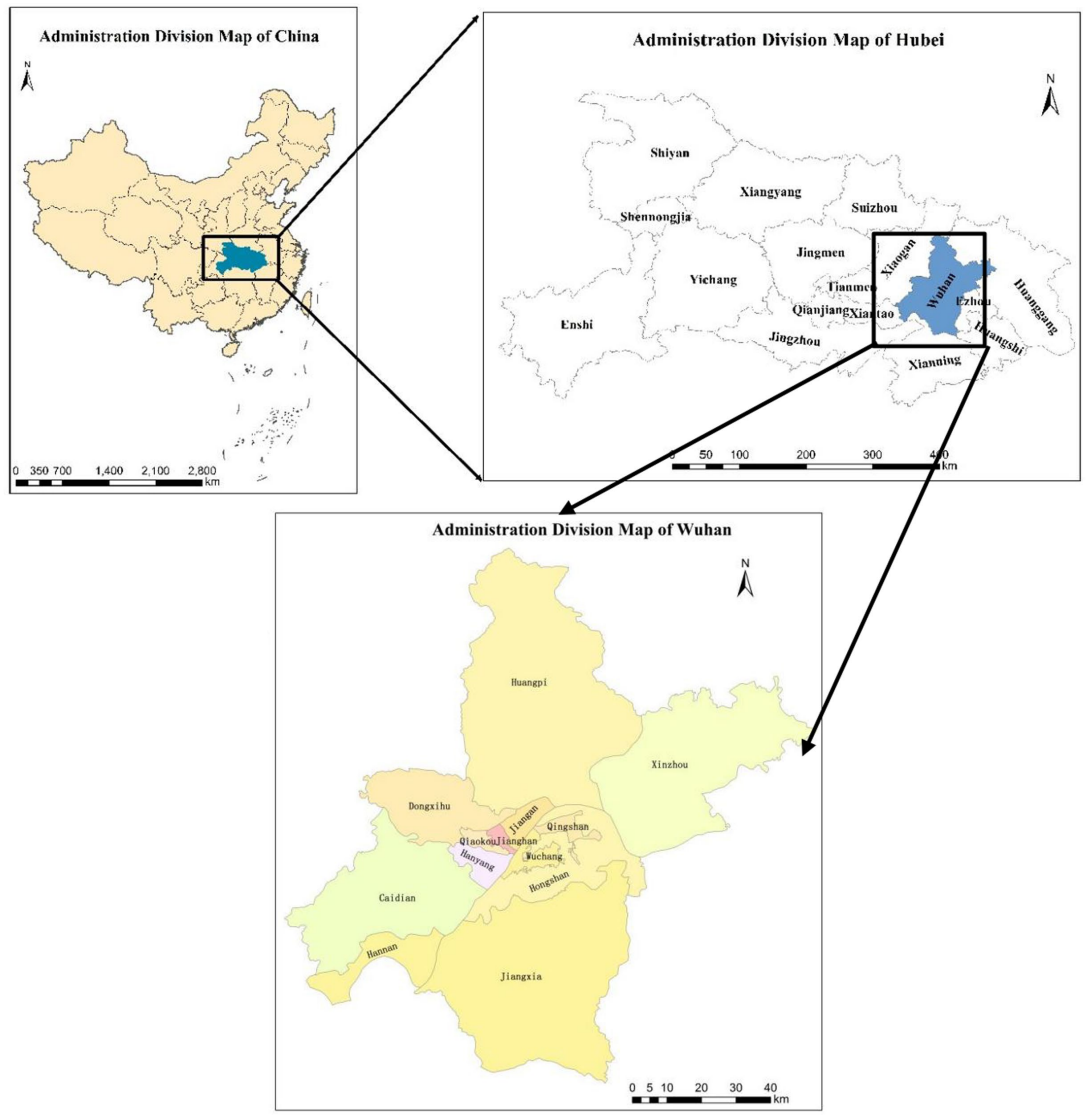
Location map of the study area, Wuhan City, PRC.

Wuhan is chosen as the research example for the reason that it is the city with better and already successful urban epidemic control worldwide. As displayed in Figure 1, Wuhan, the capital of Hubei province, is not only the core city of China and the Yangtze River Economic Belt, but also the only sub-provincial city in the six provinces of central China, which is the heart of the central urban circle. Besides, Wuhan is of great convenience for transportation, it is not only the largest water, land, and air transportation hub in China, but also the core shipping center in the middle reaches of the Yangtze River. Apart from that, its high-speed railway network radiates to more than half of China, and is the only city in central China that flies directly to the other five continents of the world. What’s more, the region with the highest economic density in China, and the heart of the Wuhan urban circle is known as the “Oriental Chicago” (52, 53). This city had 13 districts under its jurisdiction by 2019 with a total area of 8,569.15 square kilometers, a registered population of 9,083,500, and a floating population of 5,103,000. In addition, the GDP of Wuhan in 2018 has reached 1.48 trillion yuan.

### 2.2. Data

#### 2.2.1. Point of interest data of epidemic prevention facilities

According to the name of the epidemic prevention facilities publicized by the epidemic prevention headquarters of Wuhan as the key words, the POI data on Baidu Map^1^ was captured, and 39 POI points of the epidemic prevention facilities in the study area were extracted through definition, screening, weight removal, deviation correction, and spatial matching. This study divided the epidemic prevention facilities in Wuhan into 2 categories: basic epidemic prevention facilities and emergency epidemic prevention facilities. On one hand, the basic epidemic prevention facilities mainly include hospitals that existed before the epidemic and became the designated medical facilities after the outbreak. On the other hand, the emergency epidemic prevention facilities are the new mobile cabin hospitals and public service facilities such as the transformation of gymnasiums, schools, and conference centers into mobile cabin hospitals to meet the needs of controlling the epidemic and isolating the infected patients in the late stage of the epidemic. There are 24 basic epidemic prevention facilities and 15 emergency epidemic prevention facilities in Wuhan. In addition, each POI (point of interest) contains information including name, category, spatial location and so on, collected on February 25^th^, 2020. The specific information is shown in Table 1 below.

**Table 1:** Information on epidemic prevention facilities in 13 administrative regions of Wuhan.

Administrative district	Basic epidemic prevention facilities	Emergency epidemic prevention facilities
Caidian district	Tongji hospital Sino-French New Town Hospital; Western District of Union Medical College Hospital; Caidian district Maternal and Child Health Hospital	Huoshenshan Hospital
Hannan district	Hannan District Hospital of Traditional Chinese Medicine	————
Hanyang district	The Fifth Hospital of Wuhan; Wuhan Traditional Chinese Medicine Hospital (Hanyang Hospital District)	Wuhan Sports Center; Wuhan International Convention and Exhibition Center
Hongshan district	Wuhan No.3 Hospital (Guanggu Guanshan Hospital); The Second Staff Hospital of WISCO; Hubei 672 Hospital of Integrated Traditional Chinese and Western Medicine	East Lake Scenic Area; Wuhan Iron and Steel Sports Center; Shipailing Senior Vocational High School; Optics Valley Science and Technology Exhibition Center
Huangpi district	Huangpi District Traditional Chinese Medicine Hospital	Huangpi Gymnasium
Jiangan district	Wuhan Hankou Hospital; Wuhan No.6 Hospital	Wuhan National Fitness Center
Jianghan district	Wuhan Red Cross Hospital; Houhu District, Central Hospital of Wuhan; Hubei Provincial Hospital of Integrated Traditional Chinese and Western Medicine	Wuhan gymnasium; Wuhan Convention and Exhibition Center
Jiangxia district	The Eastern Hospital of Hubei Provincial People’s Hospital; Wuhan Qiaoya Boai Rehabilitation Hospital	Leishenshan Hospital; Da Hua Shan Outdoor Sports Center
Qiaokou district	Wuhan No.4 Hospital (Gutian Hospital District)	————
Qingshan district	Wuhan No.9 Hospital	————
Wuchang district	Wuhan No.7 Hospital; Wuchang Hospital; God bless Hospital; Zijing Hospital	Wuhan Hongshan Stadium
Dongxihu district	————	Wuhan Living Room (Exhibition Center)
Xinzhou district	Xinzhou District Hospital of Traditional Chinese Medicine	————

“————” indicates that no epidemic prevention facilities in the district.

#### 2.2.2. Point of interest data of residential points

This study collected POI data on Baidu Map (see text footnote 1) using keywords of “residential neighborhood” and “apartment” in Wuhan. The POI data of 301,632 residential points were extracted through definition, filtration, weight reduction, bias correction, and spatial match.

#### 2.2.3. Statistical data

In this study, the number of infected people in Wuhan was collected from the data published by Hubei Provincial Health Committee daily from January 20th to March 3rd in 2020. The spatial distribution data of the population is collected from China Statistical Yearbook, the Wuhan statistical yearbook, and the sixth general population survey Bulletin of the People’s Republic of China.

#### 2.2.4. Road network data

The road network data used in this paper are obtained from the OpenStreetMap website. On that basis, the road network is checked and improved according to the latest map of Baidu Map to eliminate useless information and null values. What’s more, based on the processed road network, the speed of the relevant roads for automobile traffic is given: 80 km/h for expressways; 60 km/h for main roads; 50 km/h for secondary roads; and 30 km/h for tertiary roads according to the Technical Standards of Highway Engineering of the People’s Republic of China and the city closure status of Wuhan.

### 2.3. Methods

#### 2.3.1. Breakpoint regression

Breakpoint regression is a Stata-based evaluation model that is mostly used to evaluate the effects of a policy. The policy makes individuals’ probability of receiving intervention on one side of the cutoff point significantly different from the other. Since the driving variable is continuous and individuals near the cutoff point are similar and comparable, so the difference in outcomes between individuals on both sides can be attributed to the policy intervention (54). In this study, the Wuhan COVID-19 epidemic was divided into two phases: the initial phase and the later phase. The initial phase of the epidemic in Wuhan is from January 20^th^ to February 17^th^ in 2020, during this period the basic epidemic prevention facilities are used to prevent and control the epidemic. The later phase is from February 18^th^ to March 13^th^ in 2020, during the period the emergency epidemic prevention facilities began to be used. Referring to the existing studies, the specific time threshold is taken as the breakpoint by using time as the grouping variable (55, 56), which is both the 30th day after the start of the epidemic, the probability of starting to use the emergency epidemic prevention facilities and being treated jumps from 0 to 1 (57). Therefore, 30 is regarded as the specific time threshold, selected as the grouping variable. The model of this research is as follows (58, 59).


(1) $$Y_{i}=\alpha +\beta T+\gamma D_{i}+\mu_{i}$$

Where: i is the time; Y is the number of infected people in Wuhan (taken as logarithm); T is the driving variable time; D is the assignment variable; *μ*_*i*_ is the random disturbance term. $D_{i}$ takes the value of 0 or 1: when T≥30, $D_{i}$=1; when T<30, $D_{i}$=0.

#### 2.3.2. Kernel density estimation

Kernel Density Estimation (KDE) calculates the density of regional spatial elements in its surrounding neighborhood and calculates the density of data points in the region, to show the agglomeration and distribution characteristics of spatial points by visualization. KDE, a popular spatial analysis tool, is used to reflect the data characteristics of spatial points, which not only contributes to helping researchers to observe and analyze the sparse distribution of data points, but also shows the spatial distribution based on the region (60, 61). The calculation method is as follows:


(2) $$F(x)=\frac{1}{nh}\overset{n}{\underset{i=1}{\sum }}k(\frac{x-x_{i}}{n})$$

Where *F*(*x*) is the kernel density, $k\left(\frac{x-x_{i}}{n}\right)$ is the kernel function, *n* is the number of spatial points in the region, *h* is the bandwidth, and *x* – *x*_*i*_ is the distance from the estimated point in the space to the measured point *x*_*i*_.

#### 2.3.3. Maximizing coverage range model

Based on the principle of the maximum number and range of coverage of demand points with a threshold value, this model is used to study the maximum coverage of facility points within a given threshold according to the road network data and road traffic speed at all levels when determining the facility points (62–64). This study mainly measured the coverage of the settlement by epidemic prevention facilities. The calculation method is as follows:


(3) $$F=\left\{v_{i}\left|r_{ic}\right.\leq cw_{r}v_{i}\in r\right\}\cup \left\{e_{ij}\left|r_{ic}+w_{ij}\right.\leq cw_{r}v_{i}\in r\right\}$$

Where, G=(v,e,c) is the spatial network, v is the set of network nodes, e is the set of edges, and c is the set of epidemic prevention facilities. $c_{w}$ is the impedance value, $w_{ij}$ is a path to the center $e_{ij}$ from edge $\left(v_{i},v_{e}\right)$, $r_{ic}$ is the cost of that path, and F is the set of maximum coverage of the service center.

#### 2.3.4. Overlay analysis

Overlay analysis means that through ArcGIS software, elements of different spaces are overlaid and attributes are assigned in the same coordinate system using identification, spatial connection, intersection, and other tools, and the generated new layer of spatial data contains the spatial attributes of all layers (65–67). This study mainly uses ArcGIS software to overlay the spatial distribution data of epidemic preparedness facilities, population, and road network in Wuhan, and to compare and analyze their spatial distribution characteristics. The analysis procedure and relevant data are as follows:

1. Calculating the maximum coverage range of epidemic prevention facilities based on official information and online map data, combined with the location data of epidemic prevention facilities.
2. Then, the spatial data of the residential point data and the spatial data of the maximum coverage range of the epidemic prevention facilities were overlaid to measure the data of the maximum coverage range of the epidemic prevention facilities to the residential points.
3. The population density data were visualized and graded using the natural breaks classification method. The spatial linkage and identification tool was then used to overlay the population density level data with the epidemic prevention facilities to analyze the relationship between the epidemic prevention facilities and the population density level.

## 3. Results

### 3.1. Spatial distribution of epidemic disaster prevention facilities

**Figure 2:**
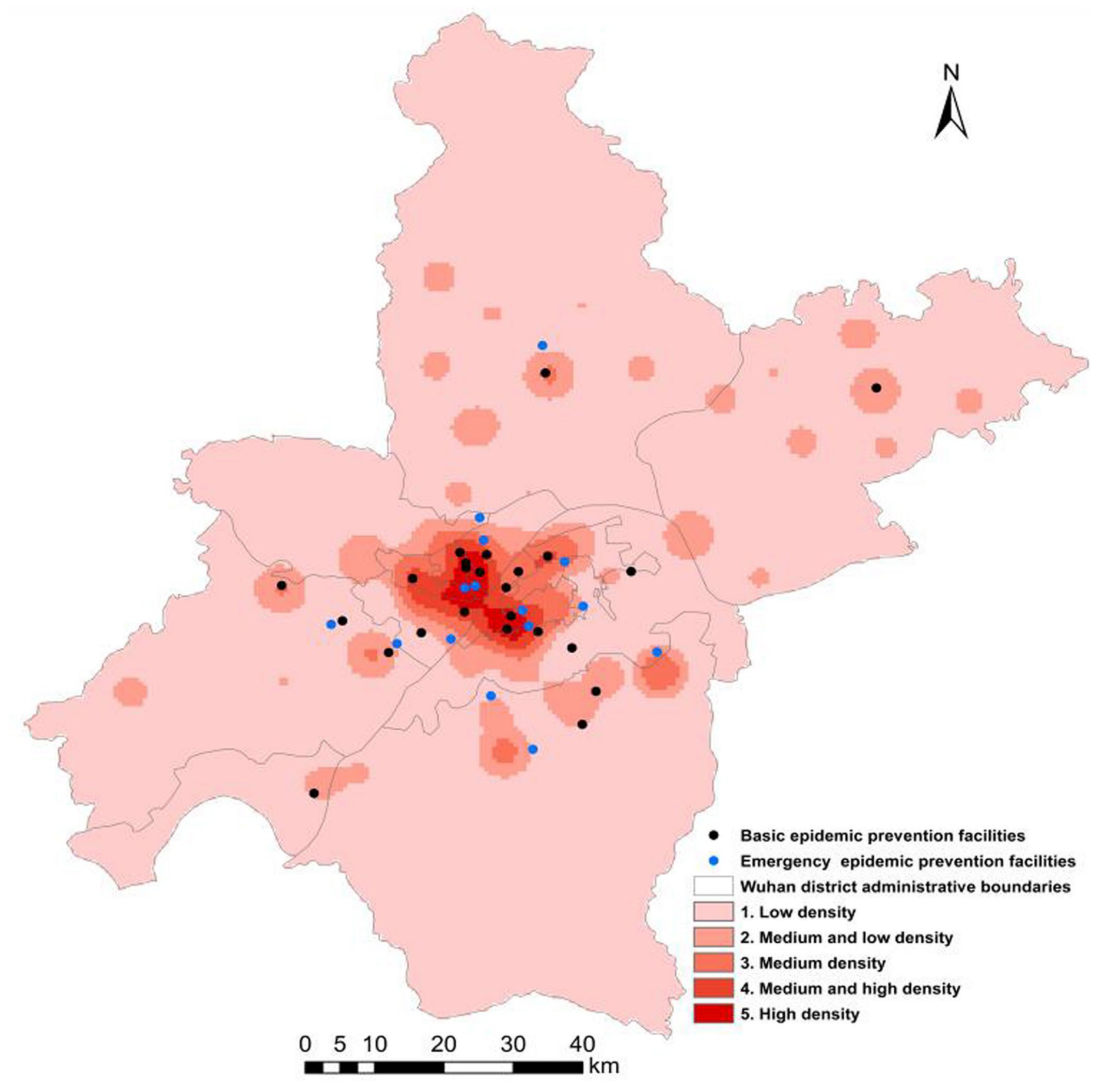
Spatial distribution of epidemic disaster prevention facilities and population density in Wuhan.

In January 2020, the COVID-19 epidemic began to spread in Wuhan. Wuhan government turned 24 basic epidemic prevention facilities into epidemic prevention facilities as designated hospitals for COVID-19 patients. Besides, 2 mobile cabin hospitals for infectious disease treatment were built, and 13 gymnasiums, conference centers, and other public infrastructure were temporarily renovated to be emergency prevention facilities. The population density was classified into five categories using the natural breaks classification method, which includes low density, medium and low density, medium density, medium, and high density and high density. And the analysis and overlay of the combination of epidemic prevention facilities and population density spatial distribution data are displayed in Table 2 and Figure 2.

**Table 2:** The number of epidemic prevention facilities and the level of population density.

Population density level	Number of basic epidemic prevention facilities	Number of emergency epidemic prevention facilities	Total
1 (Low density)	2	4	6
2 (Medium and low density)	5	3	8
3 (Medium density)	4	3	7
4 (Medium and high density)	6	2	8
5 (High density)	7	3	10

By counting the number of epidemic prevention facilities in each district of Wuhan and regarding them as the inherent relevant attributes of the region, and using ArcGIS to visualize them, we can infer the different spatial distribution of epidemic disaster prevention facilities in different regions of Wuhan, and make a preliminary statistical analysis of their spatial distribution. According to Figure 2, the spatial distribution of epidemic prevention facilities in Wuhan is basically consistent with the spatial distribution of the population, and the population is concentrated in the central urban area composed of Jianghan district, Jiang’an district, Qingshan district, Qiaokou district, and Wuchang district. As shown in Table 2, there is a positive correlation between the total number of epidemic prevention facilities and the level of population density. That is, the higher the population density, the more the epidemic prevention facilities. On one hand, the number of epidemic prevention facilities in high population density areas is as high as 10, accounting for 25% of the total number of epidemic prevention facilities. On the other hand, the number of low density population areas is the least, only 6, accounting for 12% of the total. In addition, the number of epidemic prevention facilities located in high density areas and medium and low density areas is the same. The spatial distribution of basic epidemic prevention facilities is opposite to that of emergency epidemic prevention facilities, with the spatial number of basic epidemic prevention facilities decreasing from high population density areas to low density areas. The areas with the largest number of basic epidemic prevention facilities are located in high density areas, which account for more than 30% of the total number of basic epidemic prevention facilities and account for 70% of the total number of epidemic prevention facilities in high density areas. However, the number of low density areas accounts for only 0.08% of the total number of basic epidemic prevention facilities. The areas with the largest number of emergency epidemic prevention facilities are located in low density population areas, accounting for 26% of the total emergency epidemic prevention facilities, and accounting for 66% of the total number of epidemic prevention facilities in low density areas. Additionally, the numbers of emergency epidemic prevention facilities located in high density areas, medium density areas, and medium and low density areas are the same.

The area with the most epidemic prevention facilities is Hongshan district, including 3 basic epidemic prevention facilities and 4 emergency epidemic prevention facilities. Hongshan District, as one of the political and economic cores of Wuhan, has a more developed economy and a higher population density than other areas. The number of infected people there was also relatively large, so the number of epidemic prevention facilities was larger. The epidemic prevention facilities in Hongshan district are mostly distributed in the densely populated areas in the northwest of Hongshan district, where the population distribution is above medium density, and the number of epidemic prevention facilities in the region accounts for 71% of the total number of epidemic prevention facilities in Hongshan district. Besides, the number of epidemic prevention facilities in Jianghan district is second only to Hongshan district, including 3 basic epidemic prevention facilities and 3 emergency epidemic prevention facilities. Because its population density is at level 5 and The base of infected people with the highest population density is also relatively large, so it is equipped with 6 prevention facilities in order to cut the spread of the epidemic. Apart from that, Jiangxia district, as an education concentration area of Wuhan, includes many colleges and secondary schools, junior middle schools and primary schools. The distribution of population density is dense in the north and sparse in the south. The epidemic disaster prevention facilities in this region are also located in the north with 4 epidemic prevention facilities, including 2 basic epidemic prevention facilities and 2 emergency epidemic prevention facilities. Additionally, due to the low low population density or loose spatial distribution, the number of epidemic prevention facilities in the rest regions is relatively small, with only 1 or 2 epidemic prevention facilities, or even 0. For example, in Hannan district and Xinzhou district, the population space is concentrated in a small area with a level-2 population density, so there is only one epidemic prevention facility located in densely populated areas.

### 3.2. Breakpoint regression analysis of epidemic facilities on epidemic prevention and control

**Figure 3:**
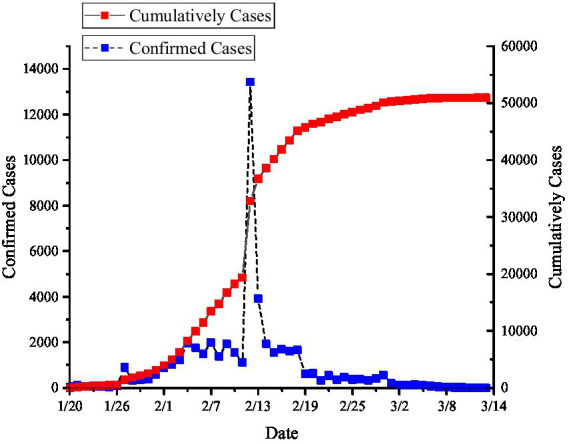
Schematic diagram of COVID-19 infection numbers in Wuhan from January 20^th^ to March 13^th^, 2020.

As shown in Figure 3, since January 20^th^, 2020, the number of confirmed cases of the epidemic in Wuhan gradually has begun to increase, but the number of basic epidemic prevention facilities was far from meeting the needs of epidemic treatment. Moreover, most of the basic epidemic prevention facilities were concentrated in the central urban area of Wuhan with a population density above level 3. The central urban area is of convenience for residents, has a large population base with strong panic, and even has a large influx of non-residents or tourists. It has led to the overloaded operation of basic epidemic prevention facilities and overcrowding in hospitals, which greatly increased the possibility of cross-infection and human-to-human transmission. Therefore, during the period from the beginning of the epidemic to the opening of emergency epidemic prevention facilities in Wuhan on February 18^th^, as displayed in Figure 3, the number of infected people in Wuhan rose from 60 on January 20^th^ to 1,600 on February 17^th^. What’s worse, the total number of infected people reached a peak of 43,468 in the initial phase, which was 724.5 times that of January 20^th^, and the number of infected people showed an upward trend of exponential growth. In the initial phase, the situation of COVID-19 infection in Wuhan was not well controlled, because there was a shortage of basic epidemic prevention facilities such as the tension of beds and the lack of medical workers.

As a result, the capacity of basic epidemic prevention facilities cannot meet the needs of a large amount of patients hospitalized, and patients were selectively treated, priority being given to patients with a critical illness. For patients with mild illness, home isolation was recommended, which greatly increased the probability of social transmission and has a negative impact on the prevention and control of the epidemic. According to the experience of using basic epidemic prevention facilities for treatment, epidemic prevention and control and the suppression of the number of infections in Wuhan, the existing urban basic epidemic prevention facilities cannot meet the requirements of urban epidemic prevention, and urban basic epidemic prevention facilities are located in densely populated areas. So the containment effect of these facilities on the epidemic is not sufficient and home isolation cannot stop the spread of epidemic.

**Figure 4:**
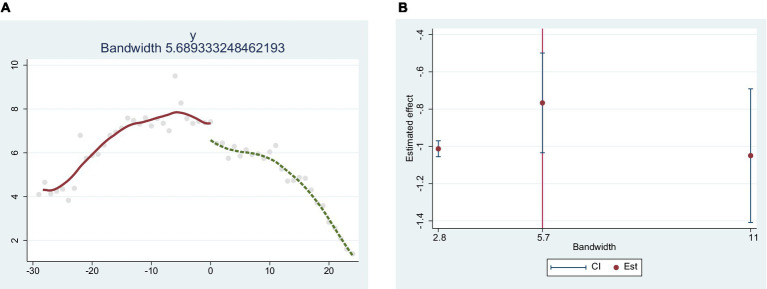
Breakpoint regression between emergency disaster prevention facilities and the number of infected people in Wuhan. **(A)** is breakpoint regression; **(B)** is bandwidth check.

After February 17^th^, Wuhan began to use emergency epidemic prevention facilities, and quickly had the infected people centralized in the mobile cabin hospital to receive treatment. As a result, the scale of the spread of the epidemic subsequently stabilized with an overall trend of containment. In order to demonstrate the existence of a causal relationship between epidemic control and the activation of emergency epidemic prevention facilities in Wuhan, this study chose the breakpoint regression method to analyze the change in epidemic scale in Wuhan. Firstly, determine the existence of breakpoints and their possible locations by graphs, then calculate and test the intercept by the default triangular kernel parameters and bandwidth. As displayed in Figure 4, the epidemic data of Wuhan from January to March, 2021 during the epidemic period was used to make a breakpoint regression graph, and it was found that there was a sharp increase in the number of infected people in Wuhan on February 18^th^, 2021, which was determined to be the location of the breakpoint, so the breakpoint regression graph was formed. It can be seen that the number of infected people decreases significantly at the breakpoint with the intercept decreasing from approximately 7.4 to 6.4, which indicates that the local treatment effect of the implementation of the policy in emergency epidemic prevention facilities is about 1. Therefore, there is a clear causal relationship between the control of the epidemic and the activation of emergency epidemic prevention facilities in Wuhan.

The significance of the breakpoint regression analysis results strictly depends on the model settings, of which the bandwidth selection is the more critical setting. Generally speaking, the smaller the bandwidth is, the smaller the influence of the omitted variables on the dependent variable will be, and the more accurate the identification of jumps will be, which makes it more accurate for the identification of jumps. Therefore, not only can it estimate the treatment effect at the breakpoint more accurately, but also mitigate the estimation bias caused by endogeneity. However, if the bandwidth is too small, it will cause an excessive loss of sample size, thus generating bias in the result. Therefore, according to the sample size and optimal bandwidth, this research takes minimizing the mean squared error ad the criterion for the selection of the optimal bandwidth, choosing 50, 100 and 200% as the bandwidth of the regression model. The regression results show that the choice of bandwidth does not have a significant impact on the regression results, and the causal relationship between the number of infections and the establishment of emergency epidemic preparedness facilities does not change significantly under different bandwidth choices. Therefore, there is a significant influence of emergency epidemic facilities on the infected people. The results revealed that bandwidth selection did not have a significant effect on the regression results, and the causal relationship between the number of infected people and the establishment of emergency epidemic preparedness facilities did not change significantly under different bandwidth selections. Therefore there was a significant effect of emergency epidemic prevention facilities on the number of infected people.

### 3.3. Analysis of the accessibility of epidemic prevention facilities

**Figure 5:**
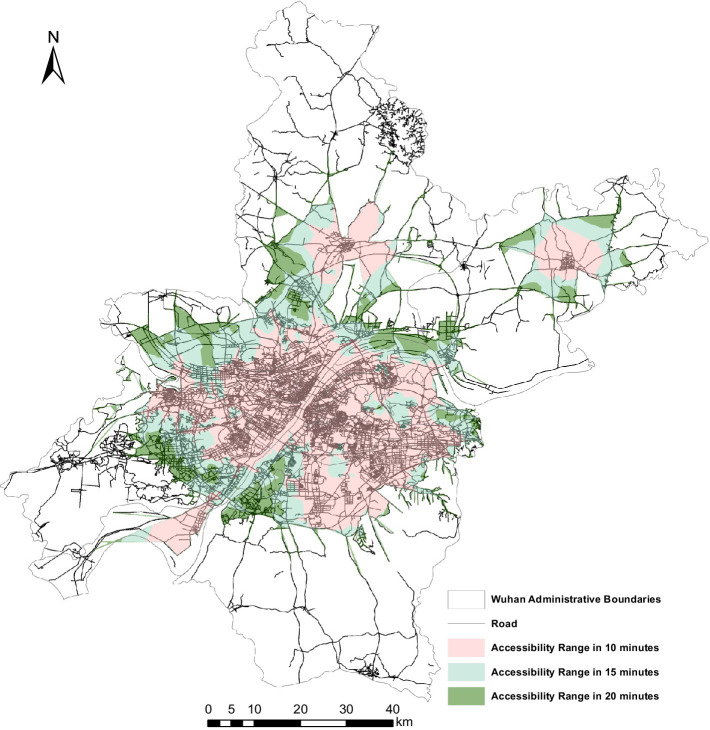
Accessibility of epidemic prevention facilities in Wuhan.

Epidemic prevention facilities, the guarantee of urban epidemic control, is not only related to the safety of people’s lives and properties, but also the main control means of urban epidemic prevention and control. Based on the fact that Wuhan has successfully interrupted the spread of the epidemic and has no infected patients at present, it is of great practical significance to analyze its epidemic prevention facilities and policy tool to combat the epidemic. This paper analyzes the service area network with the road length from the road network data to the epidemic facilities, road class and current time as the main factors. By using ArcGIS software, the arrival time to the epidemic prevention facilities in the region was divided into three levels based on the calculation of the travel time: within 10 min, within 15 min and within 20 min. According to the requirements of China’s relevant emergency regulations and the Law on the Prevention and Control of Infectious Diseases, this research believes that the 15-min accessibility time range is the most appropriate time point in public health safety emergencies, as shown in the Figure 5.

From Figure 5, there is a significant spatial difference in the accessibility of the epidemic prevention facilities in the research area, which presents a spatial distribution of a ring-shaped circle spreading from the center to the surrounding area, and the geographical distance from the central urban area was attenuated. The longer the geographical distance from the central urban area to the epidemic prevention facilities, the lower the accessibility of the epidemic prevention facilities. The areas with high accessibility are mostly concentrated in the central urban area which consists of Jianghan district, Jiangan district, Wuchang district, Hongshan district and Qiaokou district. And the road network in the central urban area is relatively dense with higher accessibility. What’s more, the accessibility of Wuhan as a whole has a clear time break, which has a dense road network and high accessibility. Within 10 min of accessibility is the largest range, followed by within 15 min of accessibility, and the smallest range is within 20 min of accessibility. Additionally, there are also a large number of areas in Wuhan where the travel time to the epidemic preparedness facilities exceeds 20 min.

The central urban areas consisting of Jianghan district, Jiangan district, Wuchang district, Hongshan district, and Qiaokou district shows high accessibility to the epidemic prevention facilities, and the travel time to the epidemic prevention facilities in central urban areas is within 10 min. Under the guidance of Wuhan’s polycentric development policy, although Caidian district and Huangpi district are far from the main urban area, they have formed central urban areas within their own administrative areas with a higher road network density and better infrastructure, so the accessibility of epidemic prevention facilities in their central urban areas within their administrative areas is also within 10 min. The areas with accessibility within 10 to 15 min to the epidemic prevention facilities are in the periphery of the central urban area, including the central urban area consisting of Jianghan district, Jiangan district, Wuchang district, Hongshan district and Qiaokou district, and the central urban areas in Caidian district and Huangpi district within their own administrative areas. Apart from that, the areas with accessibility within 15 to 20 min to the epidemic prevention facilities are mostly the junction of the district-level administrative regions, such as the junction areas of Hanyang district and Jiangxia district, Caidian district and Hannan district; the junction areas of East and West Lake district and Qiaokou district, Jianghan district and Jiangan district; and the junction areas of Hongshan district and Qingshan district. Finally, the areas with accessibility exceeding 20 min to epidemic prevention facilities are mostly located in the peripheral areas of Wuhan where the economy and transportation are not developed, including Huangpi district, Xinzhou district, Jiangxia district, Caidian district and Hannan district.

**Figure 6:**
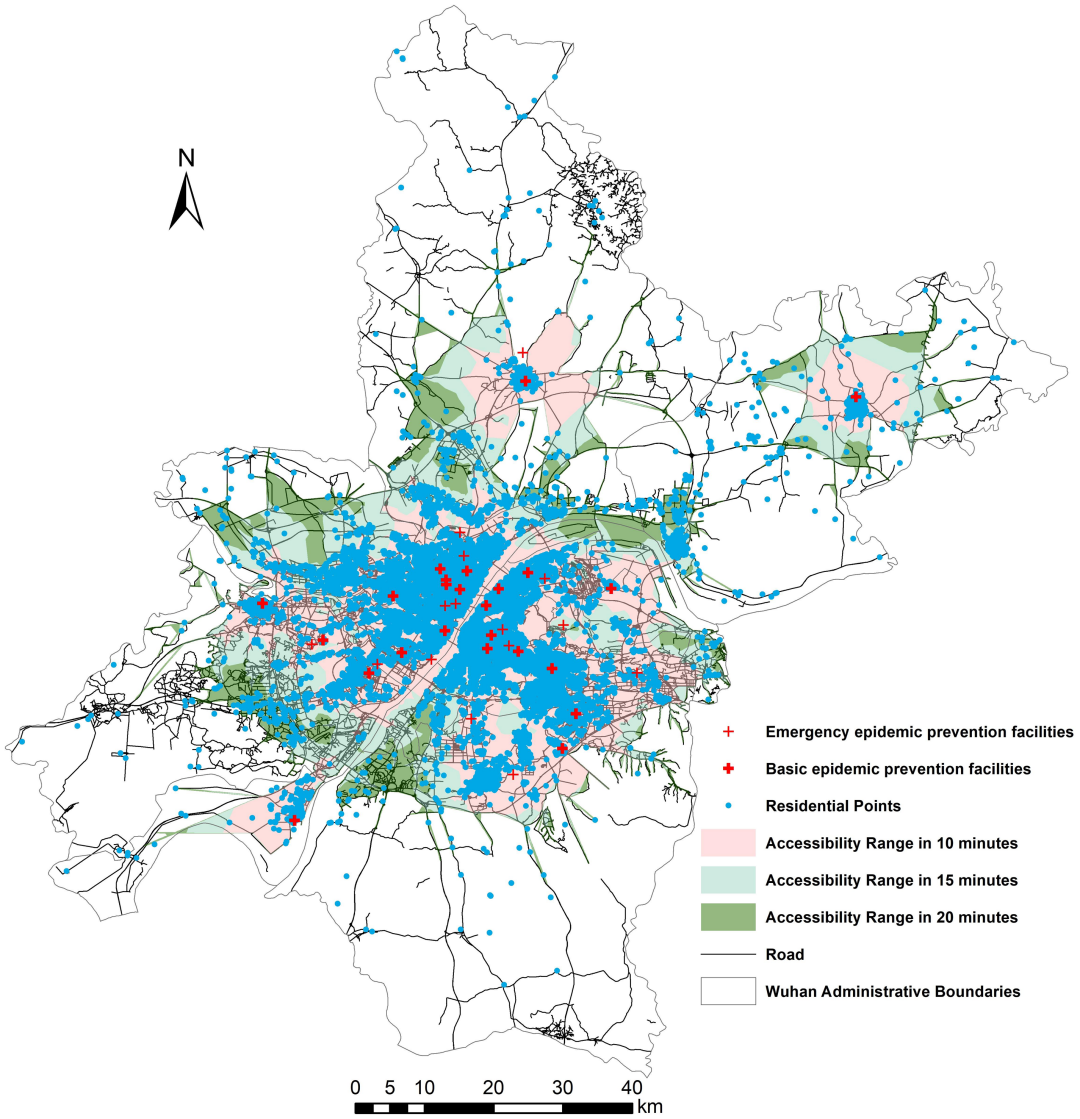
Analysis of the accessibility of epidemic prevention facilities and the overlay of residential sites in Wuhan.

As shown in Figure 6, the overlay analysis of all the residential points in Wuhan indicates that although the accessibility of the epidemic prevention facilities in Wuhan has an obvious difference and clear fault, the accessibility of the residential points to the epidemic prevention facilities is generally better. 67.3% of the residential points can arrive at the epidemic prevention facilities within 15 min. Of these, 34.2% could reach the corresponding epidemic prevention facility within 10 min, 33.1% could reach the corresponding epidemic prevention facility within 10 to 15 min, and 32.4% could reach the corresponding elderly institution within 15 to 20 min. And only 0.1% of the residential points took more than 20 min. It can be found that the accessibility of Wuhan’s residential points to the epidemic prevention facilities is higher, indicating their higher ability to isolate, control, and transport public health emergencies.

## 4. Discussion

### 4.1. Analysis of the accessibility of epidemic prevention facilities

In the overload state of basic epidemic prevention facilities, Wuhan activated the Huoshenshan Hospital located in the northeast of Caidian district, and the Leishenshan Hospital located in the north of Jiangxia district, respectively. The infected patients were centralized to receive treatment and isolation in mobile cabin hospitals which were converted from the gymnasiums, conference centers and schools. As presented in Figure 3, in addition to the outlier on February 12^th^, there was a spike in the number of confirmed patients and a peak of infected people, which was due to the simplification of medical procedures to improve the diagnosis rate. What’s more, since the epidemic began, there was a sharp drop in the number of infected people on January, 17^th^, with a decrease from 1,600 on February 17^th^ to 4 on March, 13^th^. Therefore, by making full use of the emergency disaster prevention facilities, Wuhan provided infected patients with enough space for medical isolation and treatment, which prevent the widespread of COVID-19 in families and communities.

At present, the spread of the epidemic has become a threat to urban security, and most cities do not succeed in containing the epidemic as Wuhan recently has done (51). Therefore, effective preventive and control measures should be implemented to prevent and control the spread of the epidemic based on the experience of Wuhan in controlling the COVID-19 epidemic. What’s more, future urban spatial planning should adhere to the principle of “people-oriented” in urban spatial planning (67–69). In particular, spatial planning should consider various emergency epidemic prevention facilities and sites, and reserve sufficient space and facilities for emergency epidemic prevention sites in the city to avoid passive site selection in the event of the epidemic, which may affect the control of the epidemic (70–72). For example, Wuhan temporarily does not build the Huoshenshan Hospital and the Leishenshan Hospital, but builds them in the reserved urban emergency site space, which can bring the city into epidemic control quickly and enable the epidemic to be controlled quickly (73). On the other hand, it is necessary to think about how to reuse the newly-built mobile cabin hospital and make full use of resources to avoid the waste of resources. Apart from that, the concept of health should be incorporated into urban planning in urban construction, considering the buildings’ density, population density, and other relevant factors to build urban epidemic prevention facilities in order to avoid the great impact of urban epidemics on urban development (74, 75). Future research should also consider the maximum capacity of various epidemic prevention facilities and, on the basis of that, maximize triage guidance for infected patients so as to avoid the influx of a large amount of people into epidemic prevention facilities due to panic, making various institutions overloaded or even come to a standstill, which will adversely affect the control of the epidemic (76). Only in this way can we provide a more effective urban security system for the sustainable development of the city and build a security barrier for future urban security.

### 4.2. To strengthen the mandatory urban planning for epidemic prevention facilities

Currently, cities around the world are developing urbanization significantly and most of them are in the transition period of social development (77, 78). Due to the importance of urban safety and urban disaster preparedness for sustainable urban development, most cities pay more attention to this aspect regarding it as a focus of urban planning. However, cities tend to lay much emphasis on the threat of natural disasters rather than urban epidemics. But, urban public health emergencies are second only to natural disasters in terms of casualties, and disaster prevention and control facilities for public health events such as urban epidemics are critical to sustainable urban development and urban safety (79). In addition to the number and spatial distribution of epidemic prevention facilities, the capacity and resilience of that also have a significant impact on epidemic prevention and control (12, 80). Drawing on the international experience of dealing with natural disasters from disaster-prone countries, these issues ought to be fully considered in urban planning and incorporated into mandatory regulations in order to improve urban epidemic prevention laws and regulations.

### 4.3. Research limitations

In terms of the accessibility of epidemic prevention facilities, this study only considers the resident points (81, 82). Further studies can combine the number and quality of medical facilities to conduct a comprehensive evaluation by the multiple accessibility method (18, 83–85). In addition, the influence factors selected in this article the influence factors selected in this article still need to be improved, and the analysis of them on the spatial variation of accessibility and parity of epidemic prevention facilities is not comprehensive enough. Therefore, more relevant associated factors will be selected for further analysis in future studies.

## 5. Conclusion

This study quantitatively investigated the spatial distribution, accessibility and effectiveness of epidemic prevention facilities in Wuhan by using POI data, road network data and population data. The effectiveness of the emergency epidemic prevention facilities in Wuhan was evaluated objectively and quantitatively by the breakpoint analysis method. Taking Wuhan as an example, this study summarized its spatial distribution and accessibility characteristics of epidemic prevention facilities, which provides a reference for other cities in selecting sites for urban safety and emergency prevention facilities. It is of great convenience for cities facing a certain public health emergency to quickly select from a specific candidate pool of emergency facilities (schools, gymnasiums, conference centers) for the rapid site selection and analysis with reference to study results. The system of emergency medical facilities can be established to realize the full utilization of emergency prevention facilities in the city and constitute the first line of defense for the city to respond to public health emergencies, avoiding the blind concentration of patients in large general hospitals in the city and causing medical crowding. The main conclusions of this study are as follows:

1. The spatial distribution of basic epidemic prevention facilities in Wuhan is similar to the population density, i.e., the higher the population density is, the higher the density of basic epidemic prevention facilities is. And the spatial distribution shows a clustering pattern. These results reflect that the spatial layout of basic epidemic prevention facilities in Wuhan displays saturation in the central area, insufficient supply in the periphery areas, which is inconvenient for residents in the peripheral areas, and can easily cause medical crowding in case of an epidemic outbreak.
2. This policy was analyzed by breakpoint regression. The results indicated that the opening of emergency epidemic prevention facilities had a significant contribution to the control of the epidemic. The number of infected people at the breakpoint decreased significantly with a drop of intercept from about 7.4 to 6.4, which means that the local treatment effect after the implementation of this policy was about 1. It reveals that the opening of emergency epidemic prevention facilities can effectively control the spread of the epidemic, and the emergency prevention facilities played a positive role in containing the scale of the epidemic.
3. According to the results of the accessibility analysis, the spatial differentiation of the accessibility of epidemic prevention facilities in the study area is obvious. The accessibility of epidemic prevention facilities in Wuhan showed a circular spatial distribution characteristic of spreading from the center to the surrounding area, showing the attenuation of geographical distance from the central urban area. 67.3% of residential areas could reach the epidemic prevention facilities within 15 min, and only 0.1% of residential areas had more than 20 min to reach the epidemic prevention facilities.

The analysis of the epidemic prevention facilities in Wuhan reveals that prevention facilities are of great significance to the control and containment of urban epidemics. Therefore, in the future urban construction and development, the threat of epidemic, an unexpected public health event, to urban security cannot be ignored. So epidemic prevention facilities must be incorporated into comprehensive urban disaster prevention and mitigation planning to promote cities with appropriate emergency management capabilities and improve urban resilience and security.

## Data availability statement

The original contributions presented in the study are included in the article/supplementary material, further inquiries can be directed to the corresponding author.

## Author contributions

RR: conceptualization and writing–review and editing. ZN: methodology and formal analysis. LH: data curation and writing–original draft preparation. All authors have read and agreed to the published version of the manuscript.

## Funding

This research was funded by National Natural Science Foundation of China (72074035); Graduate Research and Innovation foundation of Chongqing, China (CYS22100 and CYB22057); and Fundamental Research Funds for the Central Universities (2022CDSKXYGG006).

## Conflict of interest

The authors declare that the research was conducted in the absence of any commercial or financial relationships that could be construed as a potential conflict of interest.

## Publisher’s note

All claims expressed in this article are solely those of the authors and do not necessarily represent those of their affiliated organizations, or those of the publisher, the editors and the reviewers. Any product that may be evaluated in this article, or claim that may be made by its manufacturer, is not guaranteed or endorsed by the publisher.

## Footnotes

^1^ http://map.baidu.com/
